# Clinical Application of 3D-Printed Artificial Vertebral Body (3DP AVB): A Review

**DOI:** 10.3390/jpm14101024

**Published:** 2024-09-26

**Authors:** Roman Kiselev, Aleksander Zheravin

**Affiliations:** Meshalkin National Medical Research Centre, Novosibirsk 630055, Russia; a_zheravin@meshalkin.ru

**Keywords:** spine surgery, 3D printing in orthopedics, personalized medicine

## Abstract

**Introduction**: The choice of prosthesis for vertebral body reconstruction (VBR) remains a controversial issue due to the lack of a reliable solution. The subsidence rate of the most commonly used titanium mesh cages (TMC) ranges from 42.5% to 79.7%. This problem is primarily caused by the differences in the elastic modulus between the TMC and bone. This review aims to summarize the clinical and radiological outcomes of new 3D-printed artificial vertebral bodies (3DP AVB). **Methods**: A literature search of PubMed, Scopus and Google Scholar was conducted to extract relevant studies. After screening the titles and abstracts, a total of 50 articles were selected for full-text analysis. **Results**: Preliminary data suggest fewer implant-related complications with 3DP AVB. Most comparative studies indicate significantly lower subsidence rates, reduced operation times and decreased intraoperative blood loss. However, the scarcity of randomized clinical trials and the high variability of the results warrant caution. **Conclusion**: Most literature data show an advantage of 3DP AVB in terms of the operation time, intraoperative blood loss and subsidence rate. However, long manufacturing times, high costs and regulatory issues are this technology’s main drawbacks.

## 1. Introduction

The search for new devices for complex vertebral reconstructions is driven by the high failure rates of the currently available options. The subsidence rate of the most commonly used titanium mesh cages (TMC) is between 42.5% and 79.7% [1,2]. This problem is mainly caused by the differences in the elastic modulus between the TMC and bone. While expandable cages facilitate implantation, they do not eliminate the stress shielding effect. A meta-analysis of 26 studies found that expandable cages had a significantly lower subsidence rate compared to non-expandable cages (6% vs. 41%) [3]. However, the average displacement rate of expandable cages was 29%, which is significantly higher than that of non-expandable cages (5%). Therefore, the choice of prosthesis for vertebral body reconstruction (VBR) remains a controversial issue due to the lack of a reliable solution. A recent meta-analysis of new 3D-printed implants for VBR found a 12-times lower risk of subsidence than for TMC (OR 0.08 (95% CI: 0.03; 0,27), *p* < 0.0001). This review aims to summarize the clinical and radiological outcomes of new 3D-printed artificial vertebral bodies (3DP AVB). A literature search of PubMed, Scopus and Google Scholar was conducted to extract relevant studies. The following terms and their combinations were used in the titles/abstracts for the search: 3DP, additive manufacturing, spine, vertebral body replacement, artificial vertebral body, prosthesis, implant and cage. We included studies concerning 3DP AVB with clinical and radiological outcomes. A manual search of the references was also implemented. After screening the titles and abstracts, a total of 50 articles were selected for full-text analysis. 

## 2. Rationale

After successful experimental studies [4], additive manufacturing has found its way into clinical practice. The first insertion of a 3DP AVB in humans was described in 2016 [5]. In this case, a 14-year-old boy underwent a staged C2 spondylectomy for Ewing sarcoma. Two weeks after the en bloc removal of the tumor via a posterior approach, the remaining portion of the C2 was removed and a self-stabilizing artificial vertebral body was inserted via an anterior approach. Unfortunately, the patient died 15 months later from systemic metastases. However, neither local recurrence nor implant subsidence was observed [6]. Stable C2 reconstruction without subsidence was achieved in all nine patients studied, with a mean follow-up time of 28.6 months [6]. The reliability of the 3DP AVB was demonstrated, even without occipitocervical fixation in five patients. 

The unique anatomy of the axis (C2) and its biomechanical function hinder stable reconstruction with “off-the-shelf” implants (OTS), which explains the emergence of patient-specific implants (PSI) for C2 reconstruction. Nowadays, additive manufacturing is used for reconstruction from the atlas [7,8] to the sacrum [9,10,11]. The 3DP AVB is particularly invaluable for craniocervical reconstruction because the clivus has high angulation and there are no reliable alternatives [8]. 

Since en bloc resection is crucial for vertebral tumors, reliable anterior reconstruction is of great importance. The lack of suitable OTS devices for complex reconstructions has led to the increased use of 3DP AVB in spinal oncology. A systematic review of 19 publications confirmed the feasibility of 3DP AVB after en bloc tumor resection [12]. Complications were primarily due to tumor recurrence rather than the prosthesis itself. Subsequent reports and case series highlight the successful application of additive manufacturing in diseases such as cervical spondylotic myelopathy [13], infectious spondylitis [14], cervical tuberculosis [15], spinal deformities [16], Kümmell’s disease [17] and rheumatoid arthritis involving the cervical spine [18]. Of note, native lordosis was successfully restored using a 3DP AVB for symptomatic congenital L5 hemivertebra [7].

Implant subsidence is more common after multilevel corpectomy [19]. The main reason for failure is prolonged bone fusion due to inadequate blood supply to the surrounding tissue. Additive manufacturing has the potential to reduce the fusion time due to well-matched endplate designs and the high osseointegration potential of 3DP AVB. The first successful experience with a two-level 3DP AVB was published in 2017 [7], followed by a three-level [20] and up to six-level reconstructions [21,22]. 

## 3. Design

Selective laser sintering (SLS), selective laser melting (SLM), electron beam melting (EBM) and direct metal laser sintering (DMLS) are powder-based 3D printing techniques used for AVB manufacturing. SLS, SLM and DMLS use high-energy laser beams to melt and fuse powdered material. However, the most common technique used to produce 3DP AVB is electron beam melting (EBM), which does not require extensive post-processing. 

The preferred pore sizes for bone ingrowth are estimated to be 500–800 µm [23]. High porosity (70–90%) provides a similar Young’s modulus to cancellous bone, promoting better bone ingrowth and reducing the stress shielding effect [24]. An increase in angular mismatch between cages and vertebral endplates is known to correlate with the subsidence risk [25]. Both high porosity and conformity can be achieved with additive manufacturing. The surface of the 3DP AVB is roughened by molten titanium alloy powder particles, which facilitate cell adhesion and proliferation [26]. Therefore, the lattice structure and rough surface promote bone ingrowth even without bone grafts [27], as shown in some implants [6,20,28,29,30]. 

The high osseointegration of the prosthesis without bone grafting was confirmed in a histochemical study [31]. The 3DP AVB was removed due to the relapse of T12 osteogenic sarcoma after 14 months. Regenerated bone extending 0.5 cm into the implant and titanium struts were interconnected with a layer of chondrocytes. This confirmed ongoing endochondral bone formation more than a year after implantation. It is noteworthy that the en bloc resection was performed through adjacent vertebral bodies. As a result, the biological response of the cancellous bone was more pronounced despite its lower mechanical resistance. 

Additional elements of the 3DP AVB can also increase the stability. These include pedicle screw holes to connect the implant to the posterior instrumentation [14,32,33], an integrated posterior rod connector with an adjustable length and angles [28] and holes to guide the placement of anterior pedicle screws [15] or for fixation with an anterior plate [34]. An interesting two-piece vertebral body implant was used for recurrent L2–L3 infectious spondylitis [14]. Although there were problems in fully assembling the parts, the fusion of the bone around the device was evident after three months. 

The design of a modular vertebral prosthesis was developed by Tang et al. [35]. The vertebral prosthesis consisted of body segments and endplates of different sizes. It could be assembled with different lengths (25–200 mm, step 2.5 mm), diameters (21 mm, 24 mm and 30 mm) and degrees of curvature (0°, 4°, 8°). No instrumentation failures or dislocations were observed after the en bloc resection of spinal tumors in 26 patients. Subsidence was noted in two patients at the last follow-up, which was due to tumor recurrence and multilevel reconstruction.

The concept of a movable 3DP AVB was introduced by Zhang et al. [36]. The “ball and socket” joint consisted of an artificial mortar cup with two wings and a convex hemisphere composed of highly cross-linked polyethylene. The device was designed to maintain movement while providing stability. An animal study found that one of six movable devices was displaced six months after surgery. The average range of motion (ROM) was similar to that of the intact group, except for one goat that experienced fusion. The design of the implant was aimed at preventing disease in adjacent segments. It was shown that the movable implant, in contrast to the fusion group, did not increase the ROM in the adjacent intervertebral spaces. 

Perioperative radiation therapy is an independent predictor of prolonged fusion and rod fracture after total en bloc (TES) spondylectomy [37]. The changes in the biomechanical properties of the bone matrix after radiation therapy result in reduced fatigue resistance [38]. However, the 3DP AVB stability was not affected by either high-intensity robotic stereotactic radiotherapy [39,40] or intraoperative radiotherapy [41]. Nonetheless, artifacts in postoperative MRI scans can hinder the mapping of the target area, thereby reducing the precision and therapeutic effect. One of the possible solutions is to choose alternative materials. 

## 4. Materials 

Titanium alloy (Ti), tantalum (Ta) and polymers are used for 3DP AVB. The choice of titanium alloy Ti_6_Al_4_V is explained by its approved biocompatibility, osseointegration, load-bearing capacity and corrosion resistance [42]. The main disadvantage is radiopacity, which makes postoperative assessment difficult. Polymers do not have this problem. An experimental study showed that a 3DP polyetherketoneketone (PEKK) interbody implant had better integrity and radiographic properties than polyetheretherketone (PEEK) and Ti-coated PEEK [43]. It is noteworthy that PEKK has similar bone ingrowth properties to Ti-coated PEEK. Amelot et al. published the preliminary results of six patients who underwent anterior cervical decompression and fusion (ACCF) with 3DP PEKK for cervical myelopathy [13]. There was no deformation or change in the C2-C7 Cobb angle, in addition to significant clinical improvements. Nevertheless, the significant subsidence of one implant occurred. The authors suspected that implant misplacement was the primary cause of this subsidence. 

However, titanium alloys are believed to have higher osseointegration potential than polymer materials [44,45,46]. For example, 3DP Ti cages had better fusion rates than PEEK cages after transforaminal lumbar interbody fusion [47]. The only available clinical application of polymers for 3DP AVB production is described above. The use of polymers in additive manufacturing is limited due to their high costs and complicated production processes. 

The clinical application of 3D-printed tantalum AVB has not yet been described. A comparative in vitro study found that a 3DP Ta scaffold had the same biocompatibility in adhesion, cell viability, proliferation and differentiation as titanium scaffolds [48]. The Young’s modulus of the 3DP Ta scaffold is lower, so Ta can undergo larger deformation before fracture [49]. Therefore, tantalum has higher fatigue strength than titanium scaffolds [50]. Clinical studies are required to confirm the superiority of this material. Until then, titanium alloy remains the preferred material for additive manufacturing. 

## 5. Clinical Studies

The first clinical comparison between 3DP AVB and expandable cages found that the 3DP AVB required significantly less insertion time (<90 s vs. 46 min) and had a better endplates fit after L5 vertebrectomy [51]. A retrospective study found a lower subsidence rate and shorter fusion time for 3DP AVB implants compared to conventional TMC after TES for spinal metastases [52]. The operation time and intraoperative blood loss were also significantly lower in the AVB group. 

A biomechanical study found a non-significant difference in the primary stability and motion behavior of the thoracic spine between personalized 3DP implants and expandable devices [53]. A finite element model (FEM) showed lower C3 endplate stress with personalized 3DP AVB than with modified TMC [54]. The retrospective clinical comparison confirmed significantly fewer device-related complications with 3DP AVB [55]. The only problem was the tightening of the C1 screws, but the stability was not affected at the follow-up. To avoid this problem, the authors proposed the modification of the AVB with slanted screw holes.

The aforementioned study and eight other publications in Chinese were included in a recent meta-analysis to investigate the clinical efficacy of 3DP AVB compared to TMC after TES for spinal tumors [56]. Due to high heterogeneity, the mean differences in the operation time and blood loss were unreliable. Half of the included studies reported a shorter duration of AVB implantation, while another four studies showed no difference. Intraoperative blood loss was significantly lower during 3DP AVB reconstruction in five of eight studies. The 3DP AVB was also advantageous in terms of pain relief according to the VAS score (WMD = −0.21, 95% CI: −0.39, −0.04, *p* = 0.02), subsidence risk (OR = 0.4, 95% CI: 0.03, 0.27, *p* < 0.0001) and early complications (OR = 0.52, 95% CI: 0.29, 0.9, *p* = 0.02).

OTS implants offer the advantages of a 3D-printed structure (osseointegration and biomechanics) while overcoming the disadvantages of the manufacturing time and cost. The first described 3DP OTS AVB were anatomy-adaptive titanium mesh cages (TMC) [57]. This is an OTS implant that is available in eight sizes. The main features include a heart-shaped fornix and an angled shape in the upper and lower ends, as well as the rough surface of the implant. Therefore, the increased contact area and conformity to the endplates prevent subsidence and promote stability. Lu et al. [57] described the results of ACCF with this implant and reported no significant subsidence in 15 patients. Another retrospective series found no serious subsidence and perfect lordosis correction in 25 patients at an average follow-up time of 13.5 months after ACCF [58]. 

Dong et al. [17] conducted a retrospective comparison of 3DP OTS AVB and TMC in 28 patients with Kümmell’s disease. The patients underwent posterior vertebral column resection (PVCR), and 3DP AVB was advantageous not only in terms of the subsidence risk and local kyphotic angle correction, but also in terms of the operation time and blood loss. The time-consuming cutting of the 3DP AVB was not necessary compared to TMC. Due to the stable reconstruction, patients with 3DP AVB had better clinical outcomes as measured by the Visual Analog Scale (VAS) and the Oswestry Disability Index (ODI). 

The results of the first randomized clinical trial (RCT) were published in 2020 [34]. The study was designed as a non-inferiority comparison of the safety and efficacy of ACCF with 3DP AVB and TMC. Notably, the surgeons were blinded to the assignment until the implantation stage. TMC showed a significantly higher subsidence rate (35%) than 3DP AVB (5%). In another retrospective ACCF series, the subsidence rate was also significantly higher in the TMC group (24% vs. 10% for the 3DP AVB) [29]. Furthermore, 3DP AVB implantation required a shorter operation time.

A meta-analysis of two studies mentioned above and four prospective studies in Chinese found a seven-fold lower subsidence rate for 3DP AVB compared to TMC after ACCF (OR = 0.12, 95% CI: 0.05–0.32, *p* < 0.0001, I^2^ = 0) [59]. No significant differences were observed in the VAS and Japanese Orthopaedic Association (JOA) scores. However, the postoperative C2–C7 Cobb angle was larger after 3DP AVB implantation (weighted mean difference (WMD) = 5.88, *p* < 0.0001, I^2^ = 68%). Furthermore, 3DP AVB implantation required a significantly shorter operation time (WMD = −6.77 min, *p* = 0.04, I^2^ = 90%) with comparable intraoperative blood loss. Nevertheless, the high heterogeneity and the inclusion of non-randomized studies raise concerns about possible bias.

Wei et al. [60] provided a retrospective comparison of 3DP sacral endoprosthesis and conventional reconstructions. The design of the prosthesis was based on data from 100 patients. The patients’ CT images were used to model the process of total en bloc sacrectomy and to determine whether an endoprosthesis could be used for reconstruction. If it did not fit, a patient-specific implant (PSI) was created. The implant failure rate (10%) and implant survival (32.5 months), as well as the spinopelvic stability, were similar to those with conventional reconstruction without an endoprosthesis. Therefore, a 3DP sacral endoprosthesis is a reliable alternative to conventional sacral reconstruction. 

Lower costs and shorter manufacturing times are key advantages of OTS implants. Additionally, most 3DP AVB do not require bone grafting. This is an important advantage in spinal oncology as the use of bone autografts may be limited due to possible tumor infiltration. Zhou et a. [61] published a retrospective analysis of 23 patients after the en bloc resection of thoracolumbar tumors. They used PSIs for multilevel reconstructions in 10 patients and OTS prostheses for single-level surgeries in 13 patients. Two years after surgery, there was no significant difference in the incidence of subsidence between the implants. The overall fusion rate was 87%. However, solid fusion was achieved in all PSI cases, but in only 10 of 13 patients (76.9%) with OTS implants. 

The largest series was published by Hu et al. [62]. They used 10 PSIs and 41 3DP OTS AVB for reconstruction after the TES of thoracolumbar tumors. No subsidence was identified in the study. Of note, two postoperative PSI mismatches in the lumbar spine were reconstructed with 3DP OTS AVB. Moreover, 3DP OTS AVB may be beneficial for one-level reconstructions because it has a minimal subsidence rate and does not require bone grafting, especially in spinal oncology, where the fabrication time is a critical factor. 

An RCT was provided to compare the results of ACCF for the ossification of the posterior longitudinal ligament (OPLL) with 3DP AVB and TMC implants [63]. The study included 41 patients (21 in the 3DP AVB group and 20 in the TMC group). The 3DP AVB group required less operation time and had no swallowing difficulties compared to the TMC group. The subsidence rate was also significantly higher in the control group (4.8% vs. 40%). In contrast, the retrospective study found no difference in the subsidence rate and quality of life between 3DP OTS AVB and TMC for thoracolumbar tumor resection [64]. However, the total cost was significantly higher in the 3DP group. The low geometric conformity of the OTS design is a possible explanation for the high subsidence rate in this study (64.2%). Almost all patients who underwent radiotherapy experienced implant subsidence, which could be another explanation for the high failure rate. The third reason was osteoporosis, which was identified in 68.5% of patients. Subsidence was significantly more common in patients with osteoporosis (78% in the 3DP group and 87% in the TMC group) and after radiotherapy (89% in the 3DP group and 93% in the TMC group). Despite the assumption that sinking below the T10 level is more common [65], no difference was found between surgical levels. All results of these comparative studies are summarized in Table 1. 

## 6. Discussion

This review summarized the literature regarding the clinical and radiological outcomes of 3DP AVB with a brief description of the design and materials used for fabrication. The results of the available studies speak in favor of 3DP AVB, but, so far, there are only two randomized clinical trials (RCT) completed. Both RCTs demonstrated a significantly lower subsidence risk for 3DP AVB with OTS [34] and PSI [63] for ACCF. Due to the uniqueness of each tumor, it is difficult to provide a reliable RCT in spinal oncology. However, retrospective studies showed lower complication rates when 3DP AVB was used for TES [41,52,64]. The next major complications highlighted in the largest published series [62] were massive intraoperative bleeding, vena cava injury, deep wound infection, neurological deterioration, urine leakage, intracranial subdural hemorrhage, pneumonia and postoperative hematoma. A multivariate logistic regression analysis revealed that the lumbar spine location (OR 1.65; 95% CI: 0.26–10.38), a poor preoperative Karnofsky performance score (OR 0.92; 95% CI: 0.87–0.97) and intraoperative bleeding of more than 2000 mL (OR 9.5; 95% CI: 1.5–58.9) were the predictors of major complications. An unusual complication has been described after L2-L3 reconstruction with 3DP AVB for recurrent spondylitis. Due to suspected hypersensitivity to titanium powder particles, asymptomatic eosinophilia persisted 19 days after surgery [14].

According to a recent multicenter study [66], implant-related complications occurred in 4.6% of cases. However, most patients in the study suffered from degenerative disc disease (61.3%). The rate of implant-related complications after VBR is higher. Table 2 summarizes the available data on implant failure with 3DP AVB. After ACCF, with the exception of a PEKK implant, subsidence occurred in 3.6% (6/220), which is significantly less common than when using TMC (42.5%) [1]. The rate of implant-related complications after TES for thoracolumbar tumors was 32.4% in the largest published series [67]. According to the available data, the subsidence rate of 3DP AVB was 14.7% (29/196). However, the incidence ranged from 0% [62] to 76.9% [28]. 

Fusion rates and fusion times have not been reported in most clinical studies [29,55,56,59]. Some articles briefly mention the osteointegration potential without quantitative data [17,63]. Only a few papers state that bone fusion occurred in all cases [17,63]. Dong et al. evaluated graft fusion using the Bridwell grading criteria, but the results were not included in the paper [17]. In the randomized clinical trial [34], no difference was found in the fusion rate after ACCF (100% in the 3DP AVB group vs. 95% in the TMC group) (Wei et al., 2020). Chen et al. reported a fixation failure rate of 21.4% in 3DP AVB, while TMC failure occurred in 4.7% (*p* = 0.129) [64]. However, fixation failures included not only nonunion but also migration, screw loosening and rod breakage. Almost all failures occurred in osteoporotic patients. Only one retrospective study showed a significant advantage of 3DP AVB in terms of the fusion time after TES (10.9 ± 8.9 vs. 12.5 ± 5.2, *p* = 0.041) [52].

There are some serious limitations with 3DP AVBs. A 3DP PSI takes an average of 2 to 4 weeks to produce, limiting its use in urgent surgery. Moreover, the long manufacturing time may cause mismatches due to rapid tumor growth and massive invasion during the waiting time [41]. Therefore, a backup plan must be taken into account. Furthermore, the intraoperative strategy cannot be changed if necessary, which is particularly important in spinal oncology [30]. However, modular OTS devices overcome these disadvantages [35]. 

Another significant disadvantage of the technology is the high manufacturing costs, especially when producing multiple implants of different sizes. According to a retrospective study [64], a 3DP OTS implant was significantly more expensive than TMC with bone grafts (4.4 ± 1.3 vs. 2.6 ± 0.2 thousand US dollars, *p* = 0.001). Therefore, the total cost of hospitalization was also significantly higher (23.6 ± 5.1 vs. 18.9 ± 6.1 thousand US dollars, *p* = 0.026). Another retrospective study found that the average implant cost of 3DP OTS was nearly three times that of TMC with bone grafts (3.29 vs. 1.12 thousand US dollars, *p* = 0.001) [68]. In addition, cybersecurity related to patient data and legal regulatory issues limit the widespread use of 3D implants. Approval is usually granted on a case-by-case basis. However, the first guidelines for the registration and regulation of 3DP AVB with technical instructions have already been issued in China [69]. 

Preliminary data suggest fewer implant-related complications with 3DP AVB. However, the scarcity of randomized clinical trials and the high variability of the results warrant caution.

## 7. Conclusions

Most of the literature indicates an advantage of 3DP AVB in terms of the operation time, intraoperative blood loss and subsidence rate. The low subsidence rate is likely due to the porous structure, roughened surface and high conformity with the endplates, which provide a reduced stress-shielding effect and better osseointegration properties. Moreover, 3DP OTS implants are an efficient alternative for ACCF as they overcome the disadvantages of the manufacturing time and cost. The customization of 3DP AVB is particularly valuable for complex reconstructions in spinal oncology. However, long manufacturing times, high costs and regulatory issues remain the main drawbacks of this technology.

## 8. Future Directions

Future randomized clinical trials of OTS and PS implants are required to increase the level of evidence and address regulation issues. Additionally, long-term clinical and radiological outcomes with follow-up times exceeding five years are currently lacking. 

Preclinical studies have found that porous tantalum scaffolds exhibit superior toughness and an enhanced cushioning capacity in compression testing [70], higher fatigue stress resistance [50] and better osseointegration potential [71,72] compared to titanium alloys. The only available publication utilizing a tantalum mesh cage for vertebral body replacement involved a clinical series of 20 patients with complicated infectious spondylitis [73]. The authors reported no implant fractures or dislocations within 24 months. However, there are significant limitations associated with this material. Concerns regarding the immune response in living organisms, long-term biocompatibility, large weight, high manufacturing cost and meticulous post-processing hinder the widespread use of tantalum in spinal surgery [74]. Future animal studies and clinical trials examining the immune response and implant optimization (such as surface modification and alloying to reduce the manufacturing costs and implant weight) are needed to address these issues.

## Figures and Tables

**Table 1:** Comparative clinical studies.

Study	Year	Design	Groups (Patients)	Surgery	Main Outcomes (3DP vs. TMC)	Follow-Up, Months
C. Dong et al. [17]	2020	retrospective	3DP OTS (12) TMC (16)	PVCR for Kümmel’s disease	Operation time (164.5 ± 51.19 vs. 217.63 ± 36.29 min, *p* < 0.05) Blood loss (399.42 ± 107.98 vs. 530.31 ± 155.68 mL, *p* < 0.05) VAS score (2.67 ± 0.89 vs. 3.94 ± 1.98, *p* < 0.05) ODI score (10.92 ± 6.4 vs. 20.25 vs. 12.03, *p* < 0.05) Loss of local kyphotic angle (2.96° vs. 10.57°, *p* < 0.05) Subsidence (0.97 ± 2.23 vs. 5.39 ± 2.5 mm, *p* < 0.05)	35.5 ± 8.13 (OTS), 32.38 ± 8.13 (TMC)
F. Wei et al. [34]	2020	randomized clinical trial	3DP OTS (20) TMC (20)	ACCF	Subsidence (1.39 ± 1.05 vs. 2.39 ± 1.68 mm, *p* = 0.015) Subsidence rate (5% vs. 35%, *p* = 0.018) JOA score (16.46 ± 0.93 vs. 15.35 ± 1.81, *p* = 0.019)	6
T. Fang et al. [29]	2021	retrospective	3DP OTS (20) TMC (31)	ACCF	Operation time (106.5 ± 7.2 vs. 127.6 ± 14.4 min, *p* = 0.01) Subsidence rate (10% vs. 24%, *p* < 0.001)C2-C7 Cobb angle (22.64° ± 3.35 vs. 15.53° ± 1.86, *p* = 0.001) NDI (7.5 ± 1.52 vs. 10.17 ± 2.32, *p* = 0.04)	>12.0
H. Cheng et al. [59]	2022	meta-analysis	3DP OTS (150) TMC (164)	ACCF	Operation time (82.35 ± 21.58 vs. 84.3 ± 22.06 min, *p* = 0.04) Subsidence rate (4/120 vs. 35/134, OR = 0.12 (95% CI: 0.05, 0.32, *p* < 0.001) C2-C7 Cobb angle (22.64° ± 3.35 vs. 15.53° ± 1.86, *p* < 0.001)	12.0
Y. W. Li et al. [63]	2024	randomized clinical trial	3DP PSI (21) TMC (20)	ACCF	Operation time (50.04 ± 8.45 vs. 59.20 ± 11.95 min, *p* = 0.007) Subsidence rate (4.8% vs. 40%, *p* = 0.009) Swallowing difficulties (0% vs. 20%, *p* = 0.048)	12.0
P. Hu et al. [55]	2022	retrospective	3DP PSI (18) TMC (13)	TES (C2 tumors)	Blood loss (603 ± 132 vs. 1384 ± 232 mL; *p* = 0.008) Implant-related problems (1 vs. 4, *p* = 0.06)	>12.0
Y. Cao et al. [52]	2023	retrospective	3DP PSI (10) TMC (10)	TES (thoracolumbar metastasis)	Subsidence (1.8 ± 2.1 vs. 5.2 ± 5.1 mm; *p* < 0.006) Fusion time (12.5 ± 5.2 vs. 10.9 ± 8.9 mo; *p* = 0.041) Operation time (8.1 ± 2.3 vs. 9.1 ± 3.2 h; *p* = 0.021) Blood loss (1614.3 ± 1052.6 vs. 1850.5 ± 1116.9 mL; *p* = 0.044)	21.8 (12.0–38.0)
Z. Chen et al. [64]	2023	retrospective	3DP OTS (14) TMC (21)	TES	Subsidence rate (9/24 vs. 15/21, *p* > 0.05) Total cost (23.6 ± 5.1 vs. 18.9 ± 6.1 thousand US dollars, *p* = 0.026)	24.6 (12.0–60.0)
M. Dong et al. [56]	2024	meta-analysis of 9 retrospective studies	3DP PSI (180) TMC (194)	TES	Postoperative VAS score (mean difference: −0.21 (−0.39: −0.04, *p* = 0.02) Subsidence rate (3/68 vs. 27/72, OR 0.08 (95% CI: 0.03; 0.27), *p* < 0.0001) Early complications (29/150 vs. 48/167, OR 0.52 (95% CI: 0.29; 0.9), *p* = 0.02)	13.9

Abbreviations: 3DP—3D-printed, ACCF—anterior cervical corpectomy and fusion, JOA—Japanese Orthopaedic Association, NDI—Neck Disability Index, ODI—Oswestry Disability Index, OR—odds ratio, OTS—off-the-shelf implants, PVCR—posterior vertebral column resection, PSI—patient-specific implant, TMC—titanium mesh cage, TES—total en bloc spondylectomy, VAS—Visual Analog Scale.

**Table 2:** Subsidence rates of 3DP AVB.

Study	Year	Patients (n)	Design	Surgery	Material	Subsidence Rate (>3 mm)	Mean Follow-Up, Months
T. Lu et al. [57]	2017	15	retrospective study	ACCF	Titanium alloy, OTS	0	13.4 ± 1.4
A. Amelot et al. [13]	2018	6	retrospective study	ACCF	PEKK, PSI	16.6%	21.0 (12.0–24.0)
F. Wei et al. [34]	2020	20	RCT	ACCF	Titanium alloy, OTS	5%	6.0
T. Fang et al. [29]	2021	20	retrospective study	ACCF	Titanium alloy, OTS	10%	>12.0
H. Cheng et al. [59]	2022	120	meta-analysis	ACCF	Titanium alloy, OTS	3.3%	12.0
J. Wang et al. [58]	2022	25	retrospective study	ACCF	Titanium alloy, OTS	0	13.5 (8.0–18.0)
Y.W. Li et al. [63]	2024	21	RCT	ACCF	Titanium alloy, PSI	4.8%	12.0
J. Hu et al. [62]	2023	51	retrospective study	EBR	Titanium alloy, PSI (10), OTS (41)	0	21.0 (7.0–57.0)
M. Girolami et al. [28]	2018	13	retrospective study	TES	Titanium alloy, PSI	76.9%	10.0 (2.0–16.0)
F. Wei et al. [6]	2020	9	retrospective study	TES (C2 tumors)	Titanium alloy, PSI	0	28.6 (12.0–42.0)
X. Tang et al. [35]	2021	27	retrospective study	TES	Titanium alloy, PSI	7.4% (>2 mm)	22.0 (12.0–41.0)
H. Zhou et al. [61]	2022	23	retrospective study	TES	Titanium alloy, PSI (10), OTS (13)	21.7% (>2 mm) (10% for PSI and 30.7% for OTS)	37.0 (24.0–58.0)
Z. Chen et al. [64]	2023	14	retrospective study	TES	Titanium alloy, OTS	64.2%	24.6 (12.0–60.0)
M. Dong et al. [56]	2024	68	meta-analysis of retrospective studies	TES	Titanium alloy, PSI	4.4%	13.9
C. Dong et al. [17]	2020	12	retrospective study	PVCR	Titanium alloy, OTS	41.6% (>2 mm)	35.5±8.13

Abbreviations: ACCF—anterior cervical corpectomy and fusion, EBR—en bloc resection, OTS—off-the-shelf implants, PVCR—posterior vertebral column resection, PEKK—polyetherketoneketone, PSI—patient-specific implant, RCT—randomized clinical trial, TES—total en bloc spondylectomy.

## Data Availability

Data are available within the article.
